# Synthesis and Characterization of Phosphorus-Containing Sorbent for Basic Dye Removal

**DOI:** 10.3390/molecules28186731

**Published:** 2023-09-21

**Authors:** Monika Wawrzkiewicz, Sławomir Frynas, Beata Podkościelna

**Affiliations:** 1 Department of Inorganic Chemistry, Faculty of Chemistry, Institute of Chemical Sciences, Maria Curie-Sklodowska University in Lublin, M. Curie-Sklodowska Sq. 3, 20-031 Lublin, Poland; 2 Department of Organic Chemistry and Crystallochemistry, Faculty of Chemistry, Institute of Chemical Sciences, Maria Curie-Skłodowska University in Lublin, 33 Gliniana Str., 20-614 Lublin, Poland; slawomir.frynas@mail.umcs.pl; 3 Department of Polymer Chemistry, Faculty of Chemistry, Institute of Chemical Sciences, Maria Curie-Sklodowska University in Lublin, 33 Gliniana Str., 20-614 Lublin, Poland; beata.podkoscielna@mail.umcs.pl

**Keywords:** phosphorus-containing polymers, polymeric sorbents, basic dye adsorption, Basic Blue 3, Basic Yellow 2, Basic Red 46

## Abstract

A new phosphorus-containing sorbent was prepared by copolymerizing ethylene glycol dimethacrylate (EGDMA) and trimethylvinyl silane (TMVS) with diphenylvinylphoshine oxide (DPVO). It was characterized and applied in the removal of cationic dyes such as C.I. Basic Yellow 2 (BY2), C.I. Basic Blue 3 (BB3) and C.I. Basic Red 46 (BR46) using the batch method. Spectroscopic analysis indicated that the phosphinoyl group was introduced into the sorbent structure. Equilibrium adsorption data were fitted to the Langmuir, Freundlich, Temkin and Dubinin–Radushkevich isotherm models. The Freundlich model is the most suitable to describe the adsorption of BB3 (the Freundlich constant k_F_ = 32.3 mg^1−1/n^L^1/n^/g) and BY2 on the sorbent (13.8 mg^1−1/n^L^1/n^/g), while the Langmuir model is the most adequate to describe the adsorption of BR46 (the monolayer capacity Q_0_ = 2.7 mg/g). The kinetics of the dye adsorption follows the assumptions of the pseudo-second-order (the rate constants k_2_ = 0.087 ÷ 0.738 g/mg min) model rather than pseudo-first-order or intraparticle diffusion. The presence of Na_2_SO_4_ and cationic surfactant in the aqueous solutions inhibited dye retention by the DPVO–EGDMA–TMVS. Adsorbent regeneration efficiency does not exceed 60% using 1 M NaCl and 1 M HCl solutions in the presence of 50% *v*/*v* methanol.

## 1. Introduction

Functional synthetic polymers (FSPs) have recently received increasing interest in many applications because of their interesting properties related to, e.g., relatively easily tunable reactivity and high porosity of cross-linked FSPs [1,2]. The specific application fields of FSPs are determined by the nature of functional groups and their location and the structure of the polymer matrix [3,4].

In recent years, phosphorus-containing monomers and polymers have been the subject of extensive research. FSPs containing different phosphorus groups and cross-linked matrices are widely used in a variety of fields, ranging from nanotechnology, biotechnology, biomedical engineering, food industry, hydrometallurgy, catalysis, purification of various industrial and wastewater as polymer supports to adsorbents and ion exchange resins [5,6,7,8,9].

The first group of phosphorus polymers possess a phosphorus function in the main chain (polyphosphoesters [10] and polyphosphazenes [11]). The polymers with phosphorus present in the side part of fundamental chain (phosphonate [12] or phosphinic acid derivatives [13], fluoropolymers with phosphorus moiety [14]) are a second group. New materials with unique structures like dendrimers or hyperbranched polymers are also of great interest [15]. Diphenylvinylphosphine oxide has been polymerized by free radical copolymerization using monomers such as styrene and methyl methacrylate [16,17]. Copolymers with the addition of diphenylvinylphosphine oxide can be used as potential flame-retardant materials [18]. Many classes of porous materials have been devised in recent decades, with a great variety of chemical compositions, structural order and functions. One of them is that sorbents possess phosphorus functions inside the structure. Resins with phosphorus function were applied for the removal, preconcentration, recovery and even determination of metal ions in aqueous solutions [19,20,21,22,23,24,25]. Resins with substituted phenylphosphinic acid ligands are used in Ga(III) or In(III) sorption [19]. Magnetic nanocomposite (Fe_3_O_4_/SiO_2_/OPPh_2_) obtained by Naini et al. [20] was used as a palladium ion adsorbent. Li et al. [21] applied a phosphine-based organic framework (P-COF) for iodine capture. Islam et al. [22] prepared porous PVA/SiO_2_ composite functionalized with phosphines as nickel and manganese ion adsorbent from aqueous media of different pH. Resin consisting of silica and octyl(phenyl)-N,N-diisobutylcarbamoylmethylphoshine oxide (CMPO) (CMPO/SiO_2_-P) was used for Nd(III) sorption from a HNO_3_ solution [23] and preconcentration of selected actinide and lanthanide ions [24]. The styrene–divinylbenzene chloromethylated copolymers functionalized with iso-propylamine and diethylphosphite were examined for uptake of phenol and its derivatives from water [25]. A review of the literature reveals few reports on the usefulness of adsorbents containing phosphorus moiety for dye removal. Alosmanov et al. [26] described retention of arsenazo III toxic dye using phosphorus-based sorbent obtained by the chemical modification of butadiene rubber. 

The increasing consumption of dyes in various industries (such as textiles, paper production, plastics, paints and varnishes, cosmetics and foodstuffs) from year to year means that they are being generated into wastewater. Even small amounts of dyes in water cause problems [27]. Dyes affect the inhibition of photosynthesis in reservoirs. They exhibit toxic effects on flora and fauna and can penetrate the human food chain [28]. Most of the produced dyes are xenobiotics, i.e., chemical compounds that are foreign to the organism and not found in nature, but at the same time exhibit biological activity. Especially dangerous to aquatic ecosystems and humans are the degradation products of azo dyes, such as aromatic amines, hence the need to remove them from sewage. One technique that makes this possible is adsorption. It has many advantages over other wastewater treatment methods, as described by Akhtar et al. [28]. 

The aim of the paper was the DPVO–EGDMA–TMVS adsorbent synthesis. It was prepared by the crosslinking of diphenylvinylphosphine oxide (DPVO) with a mixture of ethylene glycol dimethacrylate (EGDMA) and trimethylvinyl silane (TMVS). Then, the material was applied as a potential polymeric sorbent for removal of basic dyes such as BB3, BY2 and BR46 from aqueous solutions. Given that literature reports on the use of such adsorbents for dye removal are rare, it seemed appropriate to prepare this type of material and evaluate its adsorption properties. The parameters such as dye concentrations, phase contact times as well as electrolytes and surfactant addition on dye retention were examined. Regeneration studies of the DPVO–EGDMA–TMVS were also investigated.

## 2. Results and Discussion

### 2.1. Synthesis of DPVO and DPVO–EGDMA–TMVS Sorbent

**Scheme 1:**
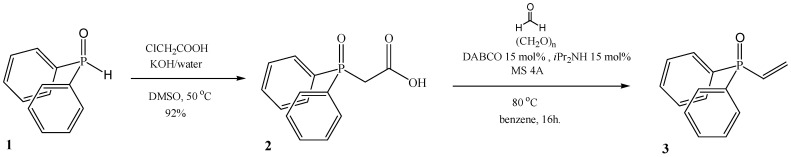
Synthesis of diphenylvinyl phosphine oxide DPVO **3**.

We previously described a functionalized polymeric sorbent based on 1,2-bis ((*E*)-2-diphenylphospinoylethenyl)-cyclohex-1-ene [29]. A phosphorus monomer containing cyclohexene ring and two ethenyl functions with phosphine oxide moieties was copolymerized with divinylbenzene. The resulting resin was used as a polymeric sorbent. In this study, we presented a polymeric sorbent based on diphenylvinylphosphine oxide **3** as a phosphorus-containing monomer. DPVO was copolymerized with a mixture of ethylene glycol dimethacrylate and trimethylvinyl silane. DPVO **3** was prepared in a two=step procedure (Scheme 1). Secondary diphenylphosphine oxide **1** reacts with chloroacetic acid in the presence of aqueous KOH solution in dimethyl sulfoxide as a solvent. After crystallization, we obtained diphenylphosphinoylacetic acid with a 92% yield. The resulting diphenylphosphinoylacetic acid **2** reacts next with paraformaldehyde in the presence of DABCO (1,4-diazabicyclo [2.2.2]octane) and *i*Pr_2_NH (diisopropylamine) as a catalyst. After column chromatography, we obtained target DPVO **3** with 85% yield.

### 2.2. Characteristics of Polymeric Sorbent

#### 2.2.1. Morphology

**Figure 1:**
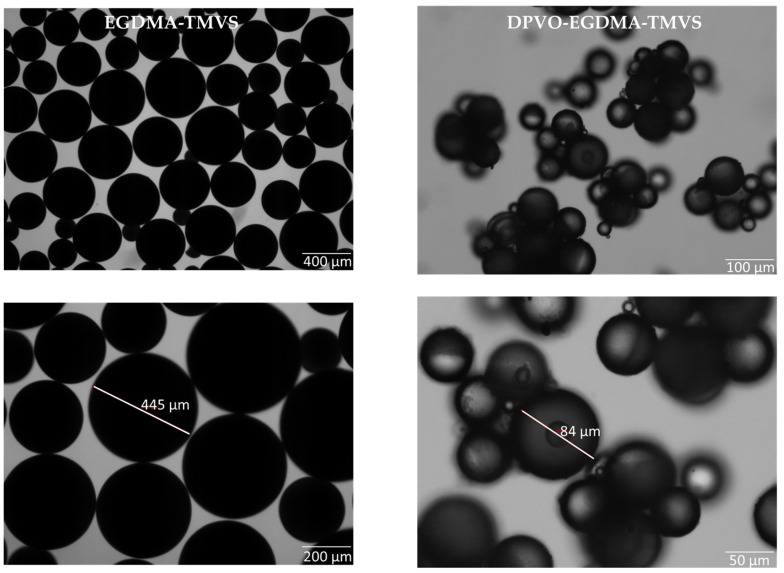
Optical images of obtained sorbent DPVO–EGDMA–TMVS.

The actual shapes of the studied microspheres are presented in Figure 1. The parent copolymers EGDMA–TMVS are characterized by a regular shape and low dispersion of microsphere rays. They have diameters in the range of 250–450 µm whereas microspheres with DPVO have diameters in the range of 50–100 µm. The addition of diphenylvinyl- phosphine oxide resulted in a significant reduction in particle sizes of the microspheres. Additionally, the material has the tendency to agglomerate.

A well-developed surface and the presence of micro- and meso-pores become important for the effective sorption processes. For this reason, parameters such as: specific surface area, pore volume and average pore diameter were established using the nitrogen adsorption method on the surface of the dry microspheres. Table 1 shows the characteristics of the porous structure of the microspheres obtained.

The EGDMA–TMVS parent material has a BET surface area of 223 m^2^/g, a total pore volume of 0.444 cm^3^/g and an average pore size of 7.754 nm. The addition of DPVO to obtain modified polymeric sorbents led to a decrease in the BET parameter to 137 m^2^/g. On the other hand, increases in the pores’ total volume (0.504 cm^3^/g) and mean size (15.029 nm) were observed. A large DPVO molecule moved the pores towards the mesopores.

#### 2.2.2. ATR–FT–IR Analysis

**Figure 2:**
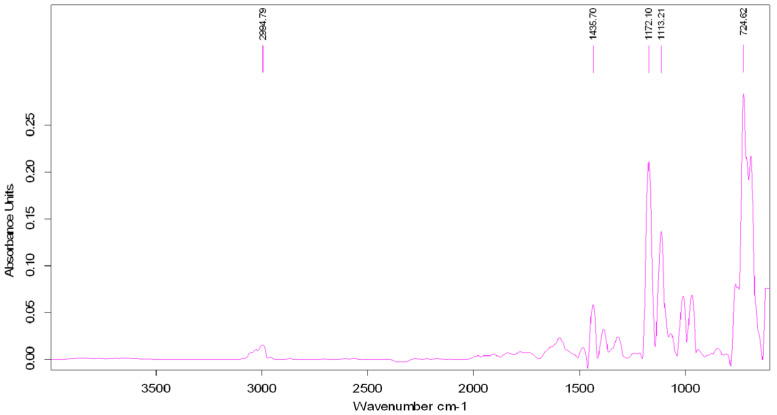
ATR–FT–IR spectra of the DPVO **3**.

Fourier-transform infrared spectroscopy with an attenuated total reflection appliance (ATR–FT–IR) was applied to record the DPVO **3** spectra (Figure 2). At 2993 cm^-1^ the C–H stretching vibrations are observed in the alkane. In the 1570 cm^−1^ region, C=C stretching vibrations appeared. The presence of the bending vibration of the C=C double bond at 985 cm^−1^ was detected. The P=O bond vibration at 1171 cm^−1^ region was noted [29].

**Figure 3:**
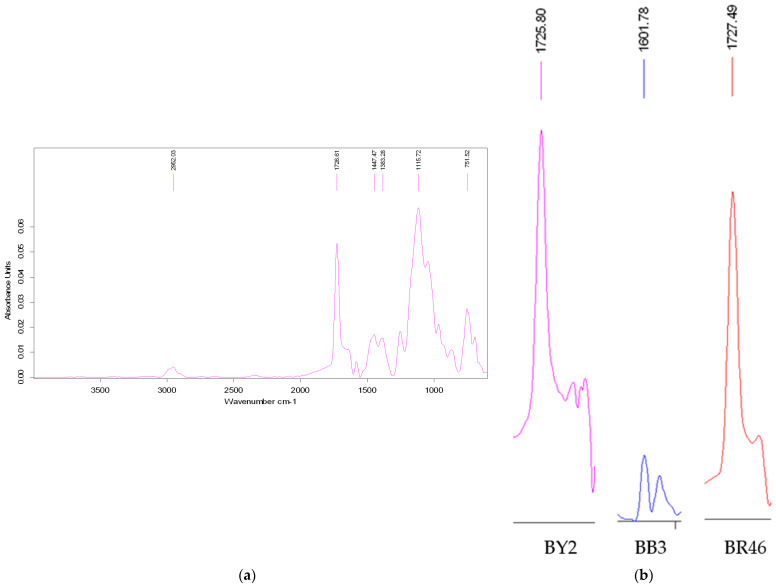
ATR–FT–IR spectra of (**a**) DPVO–EGDMA–TMVS sorbent and (**b**) selected regions after basic dye sorption.

In the ATR–FT–IR spectra of the DPVO–EGDMA–TMVS sorbent (Figure 3a), the signal from the C=C double (1578 cm^−1^) disappeared. We observed the signal of the stretching vibration of the C–H in an alkane at 2900–3000 cm^−1^ (symmetric and asymmetric stretching vibrations of methylene group). In the range of 1170 cm^−1^, the vibration of the P=O group can be distinguished.

After BB3 and BY2 sorption on the DPVO–EGDMA–TMVS, some peaks were shifted, and new ones were also detected in the ATR–FT–IR spectra. The ATR–FT–IR spectra of sorbent with BR46 dyes show no new peak. In ATR–FT–IR, BY2 signals from iminium salt at 2358 cm^−1^ were observed. After the sorption BY2, a new peak at the 1690–1700 cm^−1^ region was detected. This signal indicates the sorption of the tested dyes in iminium form. Shifted bands at the 1000–1200 and 1460 cm^−1^ regions indicate C–N stretching bonds. In the spectra of the sorbent with BB3, signals from dye at 1500–1550 cm^−1^ were observed.

#### 2.2.3. The pH of Zero-Point Charge (pH_PZC_)

**Figure 4:**
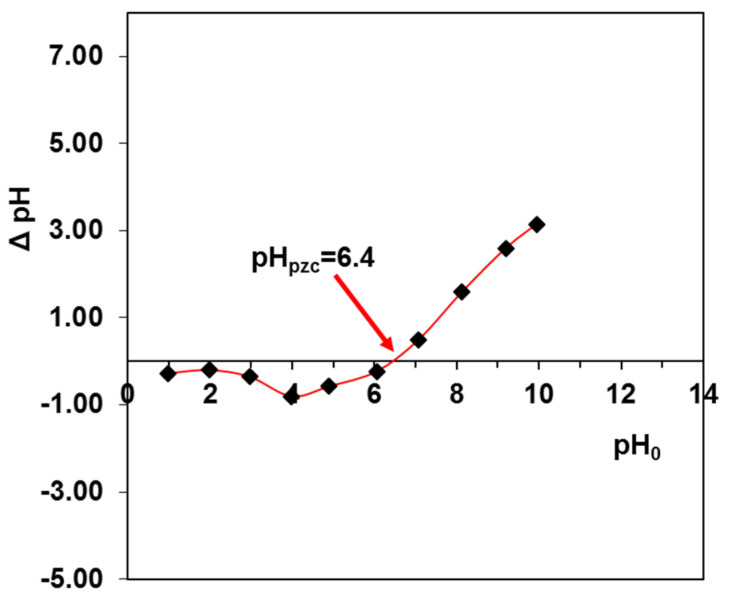
The pH_PZC_ of DPVO–EGDMA–TMVS determined using the solid addition method.

The pH_PZC_ of the obtained sorbent is a factor which determines the pH value at which its surface is electrically neutral. The pH_PZC_ of DPVO–EGDMA–TMVS was found to be 6.4 (Figure 4). At pH lower than pH_PZC_, the surface of DPVO–EGDMA–TMVS is positively charged, while at pH higher than the pH_PZC_ value the adsorbent surface has a negative charge. Therefore, the negatively charged surface of the sorbent interacts with the cationic dyes under experimental conditions.

## 3. Assessment of the DPVO–EGDMA–TMVS Adsorption Properties

### 3.1. Determination of Equilibrium Parameters

The practical use of adsorption techniques is directly related to the parameters of the structure of the adsorptive material and the conditions for conducting the process. An important parameter is the adsorption capacity (*q_e_*), the value of which makes it possible to assess the use of an adsorbent as an effective and efficient material for removing contaminants. Hence, mathematical models are constantly being sought to describe this phenomenon occurring at the phase boundary in order to determine the optimal parameters for performance of the experiment. Of the many isotherm models applied to describe the adsorption of dyes on the polymeric sorbents under equilibrium conditions, the Langmuir, Freundlich, Temkin and Dubinin–Radushkevich equations are the most commonly used:

(1) $$\frac{C_{e}}{q_{e}}=\frac{1}{Q_{0}k_{L}}+\frac{C_{e}}{Q_{0}}$$

(2) $$log\;q_{e}=log\;k_{F}+\frac{1}{n}log\;C_{e}$$

(3) $$q_{e}=\left(\frac{RT}{b_{T}}\right)lnA+\left(\frac{RT}{b_{T}}\right)lnC_{e}$$

(4) $$lnq_{e}=lnq_{m}-k_{DR}\varepsilon^{2}$$

where *q_e_*—adsorption capacity (mg/g); *C_e_*—equilibrium concentration of basic dye solutions (mg/L); *Q*_0_—the monolayer adsorption capacity (mg/g); *k_L_*—the Langmuir constant (relating to the free energy of adsorption) (L/mg); *k_F_* (mg^1–1/n^ L^1/n^/g) and 1/n—the Freundlich constants concerning adsorption capacity and the surface heterogeneity, respectively; R—gas constant (8.314 J/mol K); T—temperature (K); A (L/g) and b_T_ (J/mol)—the Temkin constants; *q_m_* (mg/g)—maximum adsorption capacity; *k_DR_* (mol^2^ J^2^)—constant related to the adsorption energy; *ε* (J/mol)—adsorption potential [30].

The isotherm parameters were calculated via linear regression using the corresponding C_e_/q_e_ vs. C_e_, log q_e_ vs. log C_e_, q_e_ vs. ln C_e_ and ln q_e_ vs. ε^2^ plots. Moreover, the mean free energy (E, kJ/mol) necessary for the removal of basic dye species from the adsorption sites to the infinity was estimated based on the Dubinin–Radushkevich isotherm applying Equation (5) [30]:

(5) $$E=\frac{1}{\sqrt{2k_{DR}}}$$

The values of determination coefficients (R^2^), chi-square (χ^2^) and residual sum of squares error (SSE) were calculated to establish the best fitting model of adsorption [30,31,32,33]. The isotherm parameters are listed in Table 2.

**Figure 5:**
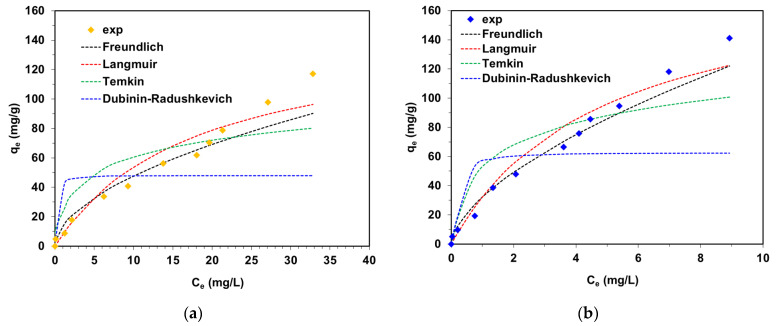

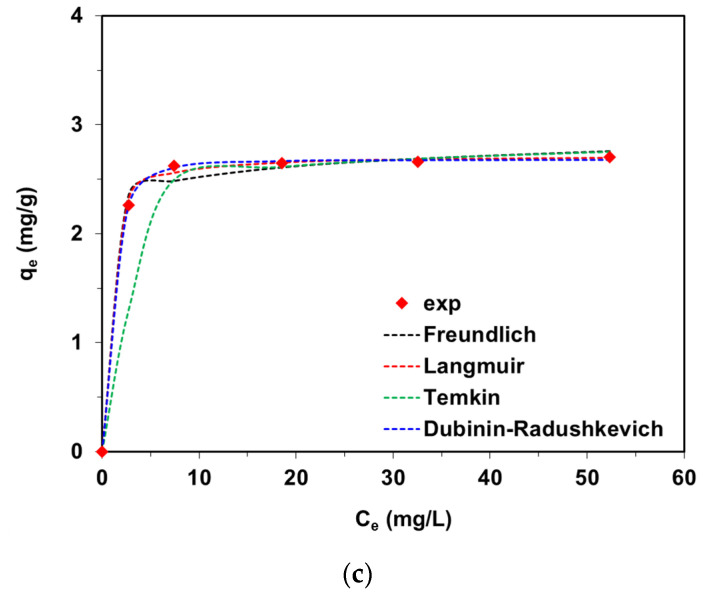
Equilibrium adsorption data of (**a**) BY2, (**b**) BB3 and (**c**) BR46 on the DPVO–EGDMA–TMVS and the fitting of the isotherm models.

Figure 5 shows the adsorption data of BY2, BB3 and BR46 on the DPVO–EGDMA–TMVS sorbent with the fitting of the experimental data to the abovementioned isotherm models.

Based on the data listed in Table 2 and presented in Figure 5, it can be concluded that the Freundlich model is the most suitable one for evaluation of BY2 and BB3 retention on the DPVO–EGDMA–TMVS. The model refers to the multilayer adsorption in the investigated experimental model on a heterogeneous surface with non-uniform energetic adsorption sites of various binding energies. The Freundlich isotherm assumed physical adsorption. The weak hydrogen bonds as well as electrostatic interactions among dye cations of BY2, BB3 and BR46 and negatively charged surface groups of the DPVO–EGDMA–TMVS under experimental conditions may be considered as the binding mechanism. The determination coefficients were found to be 0.974 and 0.938 for BB3 and BY2, respectively, and were greater than those determined for the other models. The values of χ^2^ and SSE for the Freundlich model were calculated to be 16.8 and 1166 for BY2 as well as 5.12 and 668 for BB3, respectively. The Freundlich constants k_F_ were calculated as 32.3 mg^1−1/n^ L^1/n^/g for BB3, which indicated preferential sorption compared to BY2 (13.8 mg^1−1/n^ L^1/n^/g). The 1/n parameters were less than 1, which pointed to favorable adsorption of BB3 and BY2 on the DPVO–EGDMA–TMVS sorbent. For the Langmuir adsorption model, assuming monolayer adsorption, the R^2^ = 0.999 value was greater than for the Freundlich model (R^2^ = 0.746) during BR46 adsorption on the DPVO–EGDMA–TMVS sorbent. The smallest values of χ^2^ and SSE were obtained, too. The monolayer adsorption capacity of the DPVO–EGDMA–TMVS for BR46 was equal to 2.7 mg/g. Significantly higher values of Q_0_ were calculated for BB3 (189.2 mg/g, R^2^ = 0.737) and BY2 (148.1 mg/g, R^2^ = 0.707).

The Temkin model assumed that the adsorption heat of dye molecules in the surface layer declines linearly rather than logarithmically. The b_T_ values were varied between 112.8 and 18723 J g/mol mg. However, the R^2^ values in the range 0.697–0.755 obtained for the Temkin isotherm model were smaller than for the Freundlich one.

From the Dubinin–Radushkevich isotherm model, such parameters as q_m_, k_DR_ and E were calculated. This model was proposed for the adsorption process related to volume filling of micropores. The q_m_ reflecting the maximum sorption capacities of the DPVO–EGDMA–TMVS for dyes was equal to 47.8 mg/g for BY2, 62.3 mg/g for BB3 and 2.7 mg/g for BR46. The model does not match well with the experimental data as the determination coefficients R^2^ were in the range 0.485–0.992. However, χ^2^ and SSE for BR46 adsorption were found to be 0.0005 and 0.0013, respectively. The mean free energies E were calculated in the range 1.31–3.71 kJ/mol, which exposed the physical characteristics of the basic dye adsorption on the polymeric sorbent. Unfortunately, the determination coefficients for the Dubinin–Radushkevich isotherm model are small, indicating that it does not satisfactorily describe the adsorption system.

The data summarized in Table 3 allow a reliable comparison of the equilibrium data obtained with the results already published and concerning the adsorptive removal of BB3, BY2 and BR46 dyes from aqueous solutions using adsorbents of different types.

### 3.2. Determination of Kinetic Parameters

The efficiency of basic dye sorption on the DPVO–EGDMA–TMVS as a function of time is an extremely important research step to evaluate the suitability of the adsorbate material in removal of dyes from wastewater. As the contact time between the adsorbent and adsorbate phases increases, an increase in the amount of adsorbed dyes from solutions was observed with the 10 mg/L initial concentrations of BY2, BB3 and BR46. The equilibrium state of sorption for BY2 and BB3 solutions established almost immediately (after 5 min of adsorbate–adsorbent contact time), while for BR46 it was after 20 min. The determined sorption capacities under experimental conditions for BY2, BB3 and BR46 are 9.0 mg/g, 10.0 mg/g and 4.5 mg/g, respectively. The description of the kinetics of sorption was based on the kinetic models such as pseudo-first-order (PFO), pseudo-second-order (PSO) and intraparticle diffusion (IPD) [40,41,42]:

(6) $$\log(q_{e}-q_{t})=log(q_{t})-\frac{k_{1}}{2.303}t$$

(7) $$\frac{t}{q_{t}}=\frac{1}{k_{2}q_{e}^{2}}+\frac{1}{q_{e}}t$$

(8) $$q_{t}=k_{i}t^{0.5}$$

where *q_e_* and *q_t_* (mg/g)—basic dyes amounts adsorbed at the equilibrium (known as adsorption capacities) and at specific time *t, k*_1_ (1/min)—PFO equation rate constant, *k*_2_ (g/mg min)—PSO equation rate constant, *k_i_*—intraparticle diffusion rate constant (mg/g min^0.5^).

**Figure 6:**
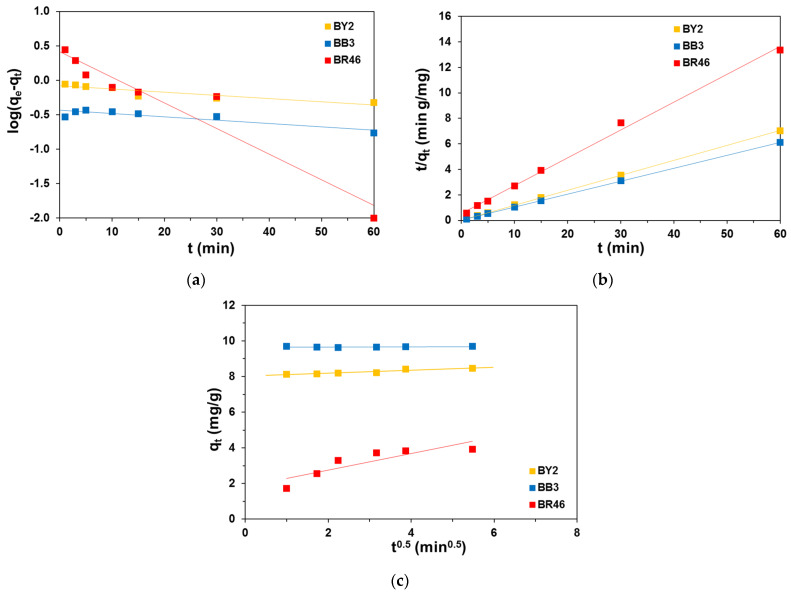
Kinetic plots for the basic dye sorption on the DPVO–EGDMA–TMVS: (**a**) PFO, (**b**) PSO and (**c**) IPD dependences.

The kinetic parameters of these models were calculated from plots presented in Figure 6 and are compiled in Table 4.

The PFO model assumed that the adsorption rate is directly proportional to the concentration difference between the amount of adsorbed substance at equilibrium and after a certain time [40]. It is generally applicable in the initial phase of the adsorption process, and the kinetics follows the PFO model when adsorption occurs through the transfer of adsorbate in the pores of the adsorbent. The values of the kinetic parameters of the PFO model calculated from the plot log(q_e_ − q_t_) vs. t (Figure 6a) and listed in Table 4 indicate that it cannot be applied to describe the experimental data. A significant discrepancy was observed between the values of the sorption capacities calculated from the model and those determined experimentally (e.g., for BY2 q_e,exp_ = 9.0 mg/g, q_e_ = 0.83 mg/g). This is also reflected in the low values of the determination coefficients (0.804 for BB3, 0.835 for BY2 and 0.925 for BR46) as well as high values of χ^2^ (67.6 for BB3, 56.6 for BY2 and 13.6 for BR46) and SSE (655.3 for BB3, 469.9 for BY2 and 47.6 for BR46).

The analysis of the obtained results indicates that the model that best describes the kinetics of the sorption of BY2, BB3 and BR46 dyes on the studied sorbent is the pseudo-second-order model. This is due to the linear course of the relationship t/q_t_ vs. t [41] (Figure 6b) and the highest values of R^2^ (0.997 for BR46, 0.999 for BY2 and BB3). Moreover, the adsorption capacity values (q_e_) calculated from the PSO model are close to the experimental values of q_e,exp_. This is also reflected in the low values of χ^2^ and SSE (Table 4).

The intraparticle diffusion model assumed that the diffusion of adsorbate in the liquid film around the sorbent is the slowest step of the process [42]. However, the plot of q_t_ vs. t^0.5^ is not a straight line passing through the origin (Figure 6c). The low values of χ^2^, SSE and R^2^ (Table 4) suggest that the intraparticle diffusion model cannot be applied for fitting of experimental kinetic data.

### 3.3. Auxiliaries Impact on Basic Dye Sorption

**Figure 7:**
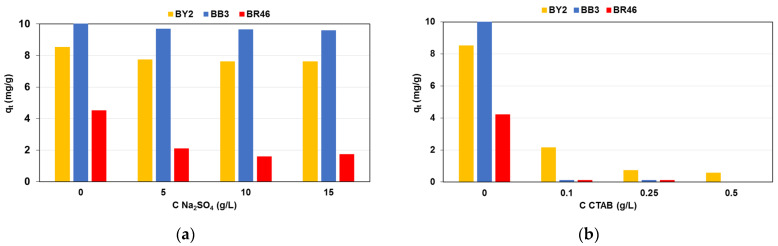
Impact of the auxiliaries on basic dye sorption on the DPVO–EGDMA–TMVS: (**a**) Na_2_SO_4_, (**b**) CTAB.

The dye sorption process can be influenced by auxiliaries (additives) such as electrolytes, surfactants, acid, bases, oxidants present in dyeing baths or effluents. In the present study, the sorption of BY2, BB3 and BR46 (C_0_ = 10 mg/L) by the DPVO–EGDMA–TMVS was studied in the presence of sodium sulfate (5–15 g/L) and cationic surfactant CTAB (0.1–0.5 g/L). Figure 7 illustrates the influence of the additives on the amounts of basic dyes uptaken after 15 min of sorption.

The basic dye uptake decreased with increasing amount of Na_2_SO_4_ and CTAB in the solution as competition between Na^+^ ions or cationic surfactant and dye cations for available adsorption sites. A similar reduction in dye sorption was observed during basic dye retention on the hybrid adsorbent based on ZrO_2_–SiO_2_ and lignin [33].

### 3.4. Regeneration Studies

The regeneration process is crucial as it determines the practicality and economic feasibility of adsorbent application in wastewater treatment and even controls the cost of the entire treatment process. DPVO–EGDMA–TMVS regeneration was performed via the chemical method using 1 M NaCl, 1 M NaOH and 1 M HCl aqueous solutions and in the presence of 50% *v*/*v* methanol (MeOH). The percentage of desorption (%D) of the basic dyes were calculated from Equation (9):

(9) $$\%D=\frac{m_{des}}{m_{ads}}100\%$$

where *m_des_*—mass of desorbed dye (mg), *m_ad_*_s_—mass of adsorbed dye (mg).

**Figure 8:**
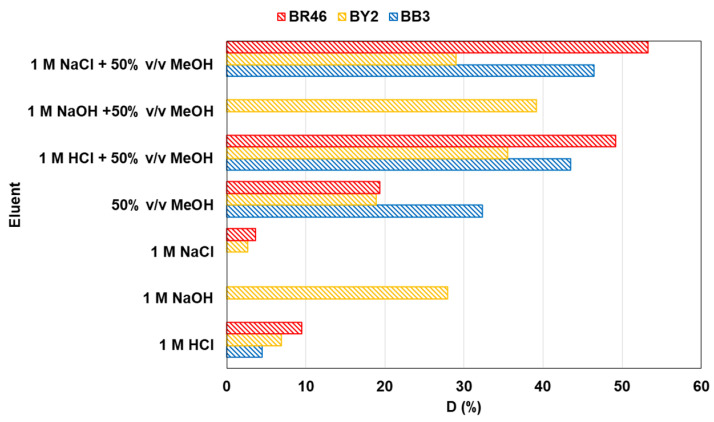
Dye desorption from the DPVO–EGDMA–TMVS.

The presence of methanol increased desorption of the dyes breaking weak physical interactions between dyes and adsorbent. This is particularly evident in the application of the 1 M NaCl + 50% *v*/*v* MeOH and 1 M HCl + 50% *v*/*v* MeOH eluents as presented in Figure 8. However, %D for does not exceed 60%.

## 4. Materials and Methods

**Figure 9:**
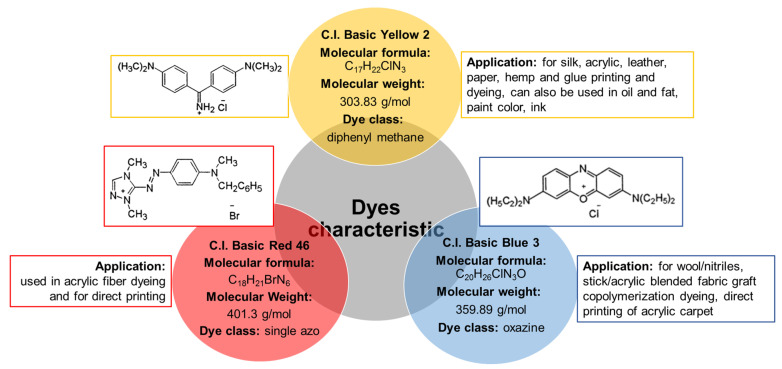
Basic dye characteristics.

Three basic dyes, namely C.I. Basic Yellow 2, C.I. Basic Blue 3 and C.I. Basic Red 46 produced by Sigma-Aldrich (Taufkirchen, Germany), were selected as adsorbates for the study. The dyes properties are presented in Figure 9. The working solutions of dyes of desired concentrations were prepared by diluting the stock solutions of the initial concentration of 1000 mg/L.

All chemicals and solvents were used without additional purification. KOH, NaOH, HCl, NaCl, Na_2_SO_4_, acetonitrile, methanol and ethanol were bought from Avantor Performance Materials Poland S.A. (Gliwice, Poland). Chloroacetic acid, DMSO, DABCO, PVA, EGDMA, TMVS, α,α-azoisobis-butyronitrile (AIBN) and diisopropylamine were ordered from Sigma-Aldrich (Taufkirchen, Germany).

Nuclear magnetic resonance (NMR) spectra were recorded on Bruker AV500 (^1^H 500 MHz, ^31^P 202 MHz, ^13^C NMR 126 MHz) spectrometers (Bruker; Billerica, MA, USA). All spectra were obtained in CDCl_3_ solutions; the chemical shifts (δ) are expressed in ppm using the internal reference to TMS and external reference to 85% H_3_PO_4_ in D_2_O for ^31^P. Coupling constants (*J*) are expressed in Hz. The abbreviations of signal patterns are as follows: s—singlet, d—doublet, t—triplet, q—quartet, m—multiplet. Mass spectra were obtained with Shimadzu LC-MS (Kinetex^®^ 2.6 μm Biphenyl 100 Å 50 × 2.1 mm LC-column, MeCN/H_2_O with HCO_2_H additive mobile phase).

The porous structure of the obtained sorbents was characterized by nitrogen adsorption at 350 °C using an ASAP 2405 adsorption analyzer (Micrometrics Instrument Corporation., Norcross, USA). Before the analysis, the materials were outgassed at 120 °C for 2 h. Specific surface areas were calculated using the Brunauer–Emmett–Teller (BET) method, with the assumption that the area of a single nitrogen molecule in the adsorbed state is 16.2 Å. Pore volumes and pore size distributions were defined by the Barrett–Joyner–Halenda (BJH) method.

### 4.1. Synthesis of Diphenylvinylphosphine Oxide (DPVO)

Diphenylvinylphosphine oxide was synthesized according to a modified procedure [43].

#### 4.1.1. Synthesis of Diphenylphosphine Oxide

Secondary diphenylphosphine oxide was prepared according to the previously described procedure [44]. Chlorodiphenylphosphine (92 g, Fluka 97%) was added to 1 N HCl (500 mL), and the mixture was stirred at room temperature overnight. Then, the mixture was extracted with methylene chloride, and the organic layer was washed with saturated aqueous NaHCO_3_ solution and saturated aqueous NaCl solution, dried over Na_2_SO_4_ and concentrated to give 74.6 g (88.5%) of diphenylphosphine oxide as a white solid of mp. 56–57 °C.

#### 4.1.2. Synthesis of Diphenylphosphinoylacetic Acid **2**

A 56% aq KOH solution (11.18 g KOH, 0.2 mol, 2.6 equiv) was added dropwise to a stirred solution of the 15.5 g (76.66 mmol) secondary diphenyl phosphine oxide **1** and 7.97 g (84.3 mmol, 1.1 equiv) of chloroacetic acid in DMSO (30 mL) at r.t. After heating for 1 h at 50 °C, the mixture was diluted with 75 mL of water. The aqueous solution was acidified with 3 M HCl, and the crude mixture was extracted with CHCl_3_. The combined CHCl_3_ extracts were dried (MgSO_4_), filtered and evaporated in a vacuum. The resulting white powder was washed with dichloromethane.

White solid; yield: 18.10 g (91%); mp 145–146 °C (Lit. [43] mp 145–146 °C).
^1^H NMR (500 MHz, CDCl_3_): δ = 7.75–7.68 (m, 4 H), 7.55–7.32 (m, 6 H), 6.81 (br s, 1 H), 3.46 (d, *J* = 14.2 Hz, 2 H).
^13^C NMR (126 MHz, CDCl_3_): δ = 167.31 (d, *J* = 5.6 Hz), 132.44 (d, *J* = 2.8 Hz), 131.07 (d, *J* = 10.3 Hz), 130.85 (d, *J* = 105.7 Hz), 128.75 (d, *J* = 12.6 Hz), 38.36 (d, *J* = 62.2 Hz).
^31^P NMR (202 MHz, CDCl_3_): δ = 30.42.

#### 4.1.3. Synthesis of Diphenylvinylphosphine Oxide **3**

A total of 18 g (69.17 mmol) of diphenylphosphinoylacetic acid **2**, 75 mL of acetonitrile, 1.16 g (10.37 mmol, 15% mol) of DABCO, 1.47 mL of diisopropylamine (10.37 mmol, 15% mol) and 1 g of MS were placed in round-bottom flask with reflux condenser equipment with a balloon and magnetic stirrer. Next, the reaction mixture was stirred at 80 °C for 12 h. The mixture was filtered, and the filtrate was concentrated under reduced pressure. Silicagel chromatography (15:1, methylene chloride/methanol) of the crude product led to the isolation of 13 g (56.9 mmol, 83%) oxide **3** as a white solid [43].

mp 115–116 °C (Lit. [43] mp 116–117 °C). ^1^H NMR (500 MHz, CDCl3): δ = 7.74–7.66 (m, 4 H), 7.57–7.50 (m, 2 H), 7.50–7.43 (m, 4 H), 6.67 (ddd, *J* = 24.6, 18.4, 12.6 Hz, 1 H), 6.39–6.20 (m, 2 H). ^13^C NMR (126 MHz, CDCl_3_): δ = 134.86, 132.22 (d, *J* = 105.0 Hz), 131.96 (d, *J* = 2.7 Hz), 131.39 (d, *J* = 10.0 Hz), 131.09 (d, *J* = 98.3 Hz), 128.62 (d, *J* = 12.2 Hz). ^31^P NMR (202 MHz, CDCl_3_): δ = 23.94. LCMS: *m*/*z* [2 M + Na]+ calculated for C_28_H_26_O_2_P_2_Na+: 479.1300; found: 479.1309.

### 4.2. Synthesis of Polymeric Microspheres

**Figure 10:**
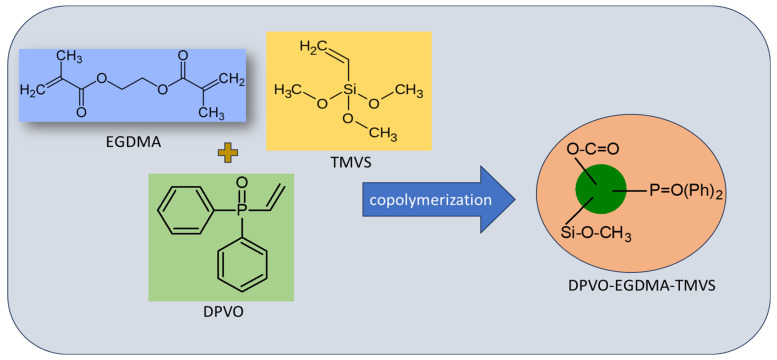
Chemical structure of monomers.

In the first stage, the phosphorous compound DPVO was dissolved in benzyl alcohol for 2 h (1 g/5 mL). The 1 g suspension stabilizer (polyvinyl alcohol, PVA) and 150 mL purified water were placed in a three-necked flask (250 mL) equipped with a mechanical stirrer, an air condenser and a thermometer. The mixture was stirred intensively to dissolve PVA for 30 min at 80 °C. Next, the ethylene glycol dimethacrylate (EGDMA) and trimetoxyvinylsilane (TMVS) were added to the previously prepared mixture DPVO with benzyl alcohol (Figure 10).

The AIBN (initiator) in the amount of 1.5 wt.% of mass monomers was added. The molar proportions of 1:1:0.5 (EGDMA/TMVS/DPVO) were applied for monomers. Additionally, parent copolymer (EGDMA + TMVS) was synthesized. The whole content was intensively mixed and appended to the aqueous phase. The stirring conditions at 80–85 °C were as follows: 350 rpm and 8 h. The prepared microspheres were filtered off then washed using distilled hot water and acetone.

The appearances and morphologies of the microspheres were studied using a Morphologi G3 Malvern optical microscope (Malvern Panalytical Ltd., Malvern, UK).

Attenuated total reflectance ATR FTIR spectra were recorded using a Bruker Tensor 27 FTIR spectrophotometer (resolution of 4 cm^−1^, 32 scans accumulated).

The pH of zero-point charge (pH_PZC_) of DPVO–EGDMA–TMVS was determined by applying the solid addition method [45]. A total of 0.2 g of DPVO–EGDMA–TMVS was immersed for 24 h in 0.01 M KNO_3_ (20 mL) of the initial pH (pH_0_) values ranging from 1.4 to 9.9 The pH was adjusted using 1 M HCl or 1 M NaOH. After 24 h, the final pH (pH_f_) of the solutions was controlled using a pH meter CP-411 (Elmetron, Zabrze, Poland). The pH_PZC_ value was determined by plotting the curve pH_0_ versus ΔpH (ΔpH = pH_0_ − pH_f_).

### 4.3. Adsorption and Desorption Tests

The batch adsorption method was used to investigate the adsorptive properties of DPVO–EGDMA–TMVS. The adsorption tests were performed at room temperature. The adsorbent mass and adsorbate volume of desired concentration used in the batch experiments equaled 0.02 g and 20 mL, respectively. The working parameters of Elpin 358+ (Elpin, Lubawa, Poland) laboratory shaker were as follows: rotary speed 180 rpm, amplitude 8. Separation of phases was achieved by filtration after predetermined time intervals (24 h for equilibrium test and 1–240 min for kinetic test). The adsorption experiments were performed in triplicate, and the mean value was calculated. For the measurement of basic dye concentrations in the aqueous phase, a Cary 60 (Agilent Techn., Santa Clara, CA, USA) UV–Vis absorption spectrophotometer was used. The assessment of the basic dyes content was performed at 430 nm for BY2, 531 nm for BR46 and 654 nm for BB3.

The regeneration of the DPVO–EGDMA–TMVS loaded with basic dyes (conditions: BB3, BY2 or BR46—10 mg/L, volume—20 mL, amount of DPVO–EGDMA–TMVS—0.02 g, time of sorption—240 min, amplitude—8, rpm—180, temperature—room) was carried out by applying the experimental conditions for regeneration: 0.02 g of DPVO–EGDMA–TMVS with loaded dyes (10 mg/g BB3, 9.0 mg/g BY2, 4.5 mg/g BR46) was put in the Erlenmeyer flasks and shaken with the solutions 1 M NaOH, 1 M HCl, 1 M NaCl, 50% *v*/*v* CH_3_OH, 1 M NaOH + 50% *v*/*v* CH_3_OH, 1 M HCl + 50% *v*/*v* CH_3_OH, 1 M NaCl + 50% *v*/*v* CH_3_OH) in the volume of 20 mL. After separation of sorbent, the basic dye concentrations were measured spectrophotometrically and calculated as the percentage of desorption (%D).

## 5. Conclusions

The paper described synthesis and characterization of a new sorbent (DPVO–EGDMA–TMVS) based on diphenylvinyl phosphine oxide, ethylene glycol dimethacrylate and trimethylvinyl silane. The sorbent beads of spherical shape have diameters in the range of 50–100 µm, surface area BET was 137 m^2^/g, the pores’ total volume was 0.504 cm^3^/g and pore mean size was 15.029 nm. The pH_PZC_ of DPVO–EGDMA–TMVS was found to be 6.4. The applicability of the obtained sorbent for the removal of basic dyes such as BY2, BB3 and BR46 was confirmed. The equilibrium state of sorption for the dye solution of the initial concentration 10 mg/L was established after 5 min of phase contact time for BB3 and BY2, while for BR46 it was observed after 20 min. Kinetic parameters calculated using different models indicate that the pseudo-second-order model (R^2^ values were in the range 0.997–0.999) can be used for description of the adsorption experiment rather than the pseudo-first-order or intraparticle diffusion. The pseudo-second adsorption rate constants k_2_ were equal to 0.087, 0.544 and 0.738 g/mg min for BR46, BY2 and BB3, respectively. Taking into account four adsorption isotherm models, the Freundlich and Langmuir ones are the most suitable to describe BY2 and BB3 as well as BR46 adsorption at equilibrium, respectively. The Freundlich isotherm model can be applied for description of BY2 (R^2^ = 0.938) and BB3 (R^2^ = 0.974) adsorption on DPVO–EGDMA–TMVS, while the Langmuir isotherm model fitted experimental data of BR46. The amounts of dyes retained by the DPVO–EGDMA–TMVS in the presence of Na_2_SO_4_ and cationic surfactant CTAB decreased in comparison to the systems without additives. Regeneration of the adsorbent using 1 M NaCl and 1 M HCl solutions in 50% *v*/*v* methanol confirmed the physical nature of the interactions between adsorbent and adsorbates. The obtained results for the removal of basic dyes using the DPVO–EGDMA–TMVS adsorbent are important both from a theoretical perspective and practical application; however, detailed economic analysis of process is required.

## Figures and Tables

**Table 1:** Porous structure properties of the obtained material.

Sorbent	Surface Area BET	Pore Total Volume	Pore Mean Size
	(m^2^/g)	(cm^3^/g)	(nm)
EGDMA–TMVS	223	0.444	7.754
DPVO–EGDMA–TMVS	137	0.504	15.029

**Table 2:** Values of parameters calculated in terms of isotherm models used.

Isotherm	Parameter	Basic Dye		
		BY2	BB3	BR46
Freundlich	$k_{F}$ (mg^1−1/n^ L^1/n^/g)	13.8	32.3	2.23
	1/n	0.53	0.61	0.05
	R^2^	0.938	0.974	0.746
	χ^2^	16.8	5.12	0.014
	SSE	1166	668	0.034
Langmuir	*k_L_* (L/mg)	0.057	0.205	2.13
	Q_0_ (mg/g)	148.1	189.2	2.7
	R^2^	0.707	0.737	0.999
	χ^2^	16.9	14.9	0.003
	SSE	969	883	0.008
Temkin	*b_T_* (J g/mol mg)	149.9	112.8	18,725
	A (L/mg)	3.87	11.1	20,059,104
	R^2^	0.697	0.745	0.755
	χ^2^	95.8	87.2	0.416
	SSE	3333	4090	0.944
Dubinin–Radushkevich	*q_m_* (mg/g)	47.8	62.6	2.7
	k_DR_ (mol^2^ J^2^)	5.15 × 10^−8^	3.62 × 10^−8^	2.86 × 10^−7^
	E (kJ/mol)	3.11	3.71	1.32
	R^2^	0.485	0.644	0.992
	χ^2^	271.9	173.5	0.0005
	SSE	11,230	12,904	0.0013

**Table 3:** Sorption capacities of different adsorbents compiled on the basis of literature review.

Basic Dye	Sorbent	Equilibrium Sorption Data	Ref.
BY2	Bagasse fly ash	Langmuir model: q_e_ = 31.18 mg/g at 30 °C, pH = 7, a.d. = 1 g/L	[31]
	poly(DVB), DVB-VBCl, poly(EGDMA), EGDMA–VBCl	Freundlich model: k_F_ = 4.56–7.85 mg^1−1/n^ L^1/n^/g at 25 °C, a.d. = 0.02 g/20 mL	[32]
	poly(DVB), CyP(Ph)4-DVB, poly(DVB)-CyP(Ph)4-DVB	Freundlich model: k_F_ = 4.56–14.2 mg^1−1/n^ L^1/n^/g at 25 °C, a.d. = 0.02 g/20 mL	[29]
	Co-participated lignin hybrid with ZrO_2_ and SiO_2_	Freundlich model: k_F_ = 11.1–45.7 mg^1−1/n^ L^1/n^/g at 20–60 °C, a.d. = 0.02 g/20 mL	[33]
	DPVO–EGDMA–TMVS sorbent	Freundlich model: k_F_ = 13.8 mg^1−1/n^ L^1/n^/g at 25 °C, pH = 8, a.d. = 0.02 g/20 mL	This study
BB3	Ethylenediamine modified rice hull	Langmuir model: q_e_ = 3.29 mg/g at 25 °C, pH = 4.7, a.d. = 0.05 g/20 mL	[34]
	Sawdust	Langmuir model: q_e_ = 28.69–27.5 mg/g at 20–50 °C, a.d. = 0.2 g/20 mL	[35]
	*Cladium mariscus* saw-sedge	Langmuir model: q_e_ = 44.29–42.07 mg/g at 20–50 °C, a.d. = 0.2 g/20 mL	
	C/SiO_2_ composite	Langmuir model: q_e_ = 925–1296 mg/g at 20–60 °C, a.d. = 0.02 g/20 mL	[36]
	poly(DVB), CyP(Ph)4-DVB, poly(DVB)-CyP(Ph)4-DVB	Freundlich model: k_F_ = 20.1–53.7 mg^1−1/n^ L^1/n^/g at 25 °C, a.d. = 0.02 g/20 mL	[29]
	DPVO–EGDMA–TMVS sorbent	Freundlich model: k_F_ = 32.3 mg^1−1/n^ L^1/n^/g at 25 °C, pH = 8, a.d. = 0.02 g/20 mL	This study
BR46	Natural bentonite from Valle del Cauca (Colombia)	Langmuir model: q_e_ = 555.6 mg/g at 25 °C, pH = 7, a.d. = 0.01 g/100 mL	[37]
	Nickel oxide nanoparticle-modified diatomite	Langmuir model: q_e_ = 105 mg/g pH = 8, a.d.= 0.005 g/25 mL	[38]
	C/SiO_2_ composite	q_e_ = 41.9–176.1 mg/g at 20–60 ^o^C, pH = 4.7, a.d. = 0.02 g/20 mL	[39]
	DPVO–EGDMA–TMVS sorbent	Langmuir model: q_e_ = 2.13 mg/g at 25 °C, pH = 8, a.d. = 0.02 g/20 mL	This study

where DVB—divinylbenzene, VBCl—4-vinylbenzene chloride, EGDMA—ethylene glycol dimethylacrylate, CyP(Ph)4—bis α,β-unsaturated phosphorylated cyclohexene, a.d.—adsorbent dose.

**Table 4:** Kinetic parameters calculated from the PFO, PSO and IPD models for basic dye sorption on the DPVO–EGDMA–TMVS sorbent from the 10 mg/L solutions.

Model	Parameter	Basic Dye		
		BY2	BB3	BR46
	q_e,exp_ (mg/g)	9.0	10.0	4.5
PFO	q_e_ (mg/g)	0.83	0.37	2.6
	k_1_ (1/min)	0.011	0.011	0.086
	R^2^	0.835	0.804	0.925
	χ^2^	56.6	67.6	13.6
	SSE	469.9	655.3	47.6
PSO	q_e_ (mg/g)	8.54	9.83	4.58
	k_2_ (g/mg min)	0.544	0.738	0.087
	R^2^	0.999	0.999	0.997
	χ^2^	0.152	0.094	0.147
	SSE	1.234	0.913	0.347
IPD	k_i_ (mg/g min^0.5^)	0.083	0.005	0.467
	R^2^	0.899	0.058	0.753
	χ^2^	0.003	0.002	0.551
	SSE	0.265	0.023	1.822

## Data Availability

The data supporting reported results can be received from the Authors.

## References

[1] Shen A., Zhang L., Xie Y., Zhu X., Hu J., Liu S. (2023). Engineering discrete synthetic macromolecules for biomedical applications. Nano Today.

[2] Chyr G., DeSimone J.M. (2023). Review of high-performance sustainable polymers in additive manufacturing. Green Chem..

[3] Makvandi P., Iftekhar S., Pizzetti F., Zarepour A., Zare E.N., Ashrafizadeh M., Agarwal T., Padil V.V.T., Mohammadinejad R., Sillanpaa M. (2020). Functionalization of polymers and nanomaterials for water treatment, food packaging, textile and biomedical applications: A review. Environ. Chem. Lett..

[4] Mane S. (2016). Functional polymers: A review. Can. Chem. Trans..

[5] Monge S., David G. (2014). Phosphorus-Based Polymers: From Synthesis to Applications.

[6] Monge S., Canniccioni B., Graillot A., Robin J.-J. (2011). Phosphorus-Containing Polymers: A Great Opportunity for the Biomedical Field. Biomacromolecules.

[7] Hiranphinyophata S., Iwasaki Y. (2021). Controlled biointerfaces with biomimetic phosphorus-containing polymers. Sci. Technol. Adv. Mater..

[8] Akhmedov V.M., Maharramov A.M., Azizov A.A., Alosmanov R.M., Bunyad-Zadeh I.A., Aliyeva S.B. (2019). Equilibrium, kinetic, and thermodynamic studies on the sorption of some heavy metal ions by the phosphorus-containing polymer sorbent. Russ. Chem. Bull. Int. Ed..

[9] Hamed F., Biji P. (2015). A novel polymer containing phosphorus–nitrogen ligands for stabilization of palladium nanoparticles: An efficient and recyclable catalyst for Suzuki and Sonogashira reactions in neat water. Dalton Trans..

[10] Chen D.-P., Wang J. (2006). Synthesis and characterization of block copolymer of polyphosphoester and poly(ε-caprolactone). Macromolecules.

[11] Allcock H.R. (2006). A Perspective of polyphosphazene research. J. Inorg. Organomet. Polym. Mat..

[12] Chen L., Wang Y.-Z. (2010). Aryl polyphosphonates: Useful halogen-free flame retardants for polymers. Materials.

[13] Kloda M., Ondrušová S., Lang K., Demel J. (2021). Phosphinic acids as building units in materials chemistry. Coordin. Chem. Rev..

[14] Wehbi M., Mehdi A., Negrell C., David G., Alaaeddine A., Ameduri B. (2020). Phosphorus-containing fluoropolymers: State of the art and applications. ACS Appl. Mater. Interfaces.

[15] Karpus A., Mignani S., Apartsin E., Zabłocka M., Shi X., Majoral J.P. (2023). Useful synthetic pathways to original, stable tunable neutral and anionic phosphorus dendrimers: New opportunities to expand dendrimer space. New J. Chem..

[16] Rabinowitz R., Marcus R., Pellon J. (1964). Synthesis, polymerization, and copolymerization of diphenyl-p-styrylphosphine, phosphine oxide, and phosphine sulfide. J. Polym. Sci. A.

[17] Rabinovitz R., Pellon J. (1961). Phosphorus-Containing Monomers. I. The Synthesis of Vinyl Phosphines, Oxides, Sulfides, and Phosphonium Compounds. J.Org. Chem..

[18] Ebdon J.R., Price D., Hunt B.J., Joseph P., Gao F., Milnes G.J., Cunliffe L.K. (2000). Flame retardance in some polystyrenes and poly(methyl methacrylate)s with covalently bound phosphorus-containing groups: Initial screening experiments and some laser pyrolysis mechanistic studies. Polym. Degrad. Stab..

[19] Trochimczuk A., Czerwińska S. (2005). In(III) and Ga(III) sorption by polymeric resins with substituted phenylphosphinic acid ligands. React. Funct. Polym..

[20] Alosmanov R.M. (2016). Adsorption of arsenazo III dye by phosphorus-containing polymer sorbent. J. Serb. Chem. Soc..

[21] Naini N., Sid Kalal H., Almasian M.R., Niknafs D., Taghiof M., Hoveidi H. (2022). Phosphine-functionalized Fe_3_O_4_/SiO_2_/composites as efficient magnetic nanoadsorbents for the removal of palladium ions from aqueous solution: Kinetic, thermodynamic and isotherm studies. Mater. Chem. Phys..

[22] Li Y., Li X., Li J., Liu W., Cheng G., Ke H. (2021). Phosphine-based covalent organic framework for highly efficient iodine capture. Micropor. Mesopor. Mater..

[23] Islam M.S., Rahaman M.S., Yeum J.H. (2015). Phosphine-functionalized electrospun poly(vinyl alcohol)/silica nanofibers as highly effective adsorbent for removal of aqueous manganese and nickel ions. Colloids Surf. A.

[24] Zhang A., Kuraoka E., Hoshi H., Kumagai M. (2004). Synthesis of two novel macroporous silica-based impregnated polymeric composites and their application in highly active liquid waste partitioning by extraction chromatography. J. Chromatogr. A.

[25] Zhang A., Wei Y., Kumagai M., Koma Y., Koyama T. (2005). Resistant behavior of a novel silica-based octyl(phenyl)-N,N-diisobutyl carbamoylmethylphoshine oxide neutral extraction resin against nitric acid, temperature and γ-radiation. Radiat. Phys. Chem..

[26] Davidescu C.-M., Ardelean R., Popa A. (2019). New polymeric adsorbent materials used for removal of phenolic derivatives from wastewaters. Pure Appl. Chem..

[27] Chauhdary Y., Hanif M.A., Rashid U., Bhatti I.A., Anwar H., Jamil Y., Alharthi F.A., Kazerooni E.A. (2022). Effective removal of reactive and direct dyes from colored wastewater using low-cost novel bentonite nanocomposites. Water.

[28] Akhtar A., Hanif M.A., Rashid U., Bhatti I.A., Alharthi F.A., Kazerooni E.A. (2022). Advanced treatment of direct dye wastewater using novel composites produced from hoshanar and sunny grey waste. Separations.

[29] Frynas S., Wawrzkiewicz M. (2023). Synthesis, characterization and application of a new functionalized polymeric sorbent based on alkenylphoshine oxide. Polymers.

[30] Murphy O.P., Vashishtha M., Palanisamy P., Vasanth Kumar K. (2023). A review on the adsorption isotherms and design calculations for the optimization of adsorbent mass and contact time. ACS Omega.

[31] Mall I.D., Srivastava V.C., Agarwal N.K. (2007). Adsorptive removal of auramine-O: Kinetic and equilibrium study. J. Hazard. Mater..

[32] Wawrzkiewicz M., Podkościelna B. (2022). Innovative polymer microspheres with chloride groups synthesis, characterization and application for dye removal. Processes.

[33] Wawrzkiewicz M., Podkościelna B., Jesionowski T., Klapiszewski Ł. (2022). Functionalized microspheres with co-participated lignin hybrids as a novel sorbents for toxic C.I. Basic Yellow 2 and C.I. Basic Blue 3 dyes removal from textile sewage. Ind. Crops Products.

[34] Ong S.T., Lee C.K., Zainal Z. (2009). A comparison of sorption and photodegradation study in the removal of basic and reactive dyes. Aust. J. Basic Appl. Sci..

[35] Bartczak P., Wawrzkiewicz M., Borysiak S., Jesionowski T. (2022). Cladium mariscus saw-sedge versus sawdust—Efficient biosorbents for removal of hazardous textile dye C.I. Basic Blue 3 from aqueous solutions. Processes.

[36] Wiśniewska M., Wawrzkiewicz M., Onyszko M., Medykowska M., Nosal-Wiercińska A., Bogatyrov V. (2021). Carbon-silica composite as adsorbent for removal of hazardous C.I. Basic Yellow 2 and C.I. Basic Blue 3 dyes. Materials.

[37] Paredes-Quevedo L.C., González-Caicedo C., Torres-Luna J.A. (2021). Removal of a Textile Azo-Dye (Basic Red 46) in Water by Efficient Adsorption on a Natural Clay. Water Air Soil Pollut..

[38] Sheshdeh R.K., Nikou M.R.K., Badii K., Limaee N.Y., Golkarnarenji G. (2014). Equilibrium and kinetics studies for the adsorption of Basic Red 46 on nickel oxide nanoparticles-modified diatomite in aqueous solutions. J. Taiwan Inst. Chem. Eng..

[39] Wiśniewska M., Chibowski S., Wawrzkiewicz M., Onyszko M., Bogatyrov V.C.I. (2022). Basic Red 46 removal from sewage by carbon and silica based composite: Equilibrium, kinetic and electrokinetic studies. Molecules.

[40] Lagergren S. (1898). About the theory of so-called adsorption of soluble substances, Kungliga Svenska Vetenskapsakademiens. Handlingar.

[41] Ho Y.S., McKay G. (1999). Pseudo-second order model for sorption processes. Process Biochem..

[42] Weber W., Morris J. (1963). Kinetics of adsorption on carbon from solutions. J. Sanit. Eng. Div..

[43] Dziuba K., Frynas S., Szwaczko K. (2021). Knoevenagel Cnondensation of Phosphinoylacetic Acids with Aldehydes: An Efficient One-Pot strategy for the Synthesis of P-functionalized Alkenyl Compounds. Synthesis.

[44] Kabat M., Garofalo L.M., Daniewski A.R., Hutchings S.D., Liu W., Okabe M., Radinov R., Zhou Y. (2001). Efficient synthesis of 1r-fluoro A-ring phosphine oxide, a useful building block for vitamin D analogues, from (S)-carvone via a highly selective palladium-catalyzed isomerization of dieneoxide to dieneol. J. Org. Chem..

[45] Wan Ngah W.S., Hanafiah M.A.K.M. (2008). Adsorption of copper on rubber (Hevea brasiliensis) leaf powder: Kinetic, equilibrium and thermodynamic studies. Biochem. Eng. J..

